# Transcriptomic insights into the healthspan-enhancing effects of *C. chinensis* seed and *E. ulmoides* bark extracts in *Caenorhabditis elegans*

**DOI:** 10.1007/s10522-025-10349-1

**Published:** 2025-11-13

**Authors:** Shimaa M. A. Sayed, Anna Pitas, Christian Schmitz-Linneweber, Nadine Saul

**Affiliations:** 1 Molecular Genetics Group, Institute of Biology, Faculty of Life Sciences, Humboldt University of Berlin, Philippstr. 13, 10115 Berlin, Germany; 2 Botany and Microbiology Department, Faculty of Science, New Valley University, El-Kharga, 72511 Egypt

**Keywords:** *Caenorhabditis elegans*, *Cuscuta chinensis*, *Eucommia ulmoides*, Traditional Chinese medicine, Aging, *far-3*

## Abstract

Healthspan, the disease-free period of life, has become a central focus in aging research. *Cuscuta chinensis* seed and *Eucommia ulmoides* bark extracts, two traditional Chinese medicine (TCM) remedies, have shown promising healthspan-extending effects in *Caenorhabditis elegans*. In this study, RNA-seq analysis of aged worms treated with these extracts revealed significant transcriptomic alterations. Gene ontology and KEGG pathway analyses indicated upregulation of genes involved in immune defense, lysosomal function, and protein homeostasis, which may underlie the shared phenotype of enhanced stress resistance and lifespan extension. Beyond these effects, *C. chinensis* further improved multiple health parameters. Consistent with its broad spectrum of phenotypes, *C. chinensis* induced extensive transcriptomic remodeling involving over 3000 differentially expressed genes. Modulating collagen-, *unc*-, and muscle-related genes may explain improved locomotion, while upregulation of *mec* genes could contribute to enhanced mechanosensation. Notably, *far-3*, encoding a fatty acid- and retinol-binding protein, was upregulated more than 150-fold, and RNA interference assays demonstrated that FAR-3 is necessary for *C. chinensis*-induced healthspan improvement. Furthermore, *C. chinensis* influenced genes linked to antagonistic pleiotropy and insulin-like signaling, suggesting a systemic, hormesis-driven reprogramming of aging processes. Together, these findings uncover both shared and distinct molecular mechanisms through which *C. chinensis* and *E. ulmoides* promote healthspan in *C. elegans*.

**Supplementary Information:**

The online version contains supplementary material available at 10.1007/s10522-025-10349-1.

## Introduction

Aging is a ubiquitous phenomenon characterized by a decline in cognitive and physical abilities, the onset of age-related diseases (ARDs), reduced stress tolerance, and overall frailty. Although commonly regarded as “natural” and therefore inherently unavoidable, aging is in fact influenced by lifestyle (Gao et al. 2018; Sanada et al. 2025) and can be modified through genetic intervention (Apfeld and Alper 2018; Kenyon et al. 1993; López-Otín et al. 2023). In an effort to prioritize quality of life over duration, a particular focus has emerged in aging research—healthspan, the period of an individual's life free from ARDs and age-related disabilities (Kaeberlein 2018; Masfiah et al. 2025). Healthspan has neither a defined start nor an end; it is a dynamic variable representing the physiology and disease mechanisms unique to each organism (Hansen and Kennedy 2016), as well as a tool to quantify the effects of genes, environmental parameters, and health interventions (Kaeberlein 2018). A key objective in aging research is to identify genetic interventions that enhance healthspan without adversely affecting lifespan.

Traditional Chinese medicine (TCM) is an herbal medical system widely used throughout Asia, with a history spanning thousands of years (Jiashuo et al. 2022). Since the 1950s, efforts to integrate TCM into Western medicine (WM) have emphasized the need for rigorous evaluation of TCM efficacy to identify treatments that genuinely improve human health (Fung and Linn 2015; Xu et al. 2013). These efforts were reinforced by the eleventh revision of the International Statistical Classification of Diseases and Related Health Problems (ICD-11) adopted by the World Health Organization (WHO) in 2019 (Harrison et al. 2021). ICD-11 acknowledges the contribution of TCM to WM and supports further rigorous testing and quality control of TCM products (Lam et al. 2019; Liu et al. 2015). A notable example of a successful TCM-derived drug is the antimalarial artemisinin, whose discovery and development earned Tu Youyou the 2015 Nobel Prize in Physiology or Medicine (Tu 2016). Nonetheless, ongoing database analyses (Fang et al. 2021) and randomized controlled clinical trials continue to explore the efficacy of TCM, and the extent of its integration into WM remains under evaluation (Hu et al. 2025).

*Cuscuta chinensis* Lam. (*C. chinensis*), a parasitic plant (Tanase et al. 2012) distributed across Africa and Asia, is one of the earliest and most widely prescribed TCM therapeutics (Donnapee et al. 2014). It has been associated with various health benefits, including protective and therapeutic effects against multiple aging-related diseases (Donnapee et al. 2014; Wu et al. 2022b; Yang et al. 2017). To distinguish genuine biological effects from potential placebo responses, studies have aimed to document and metabolically explain these claims in humans, cell cultures, and model organisms (Kang et al. 2014; Sun et al. 2014; Wang et al. 2014; Wei et al. 2019; Wu et al. 2018; Yang et al. 2011). Some studies have validated these effects, offering mechanistic insights, whereas others remain exploratory. *Eucommia ulmoides* Oliv. (*E. ulmoides*) is another important medicinal plant in TCM. Its bark, leaves, staminate flowers, and seeds are edible and valued for promoting vitality, strengthening bones, and enhancing stress resistance (Peng et al. 2024). Recent research indicates that *E. ulmoides* exhibits antioxidant, anti-inflammatory, and antidiabetic activities, along with positive effects on immune function and bone metabolism (Huang et al. 2025; Kołtun-Jasion et al. 2025; Yang et al. 2024). These properties are largely attributed to its high content of flavonoids, polysaccharides, and other bioactive natural compounds (Li et al. 2025; Liu et al. 2025).

To study aging efficiently, *Caenorhabditis elegans* (*C. elegans*) offers significant advantages over other model organisms (Roussos et al. 2023; Son et al. 2019; Tissenbaum 2015; Weinkove 2024; Zarroug 2025). *Caenorhabditis elegans* is a one-millimeter-long, self-fertilizing hermaphrodite nematode with a transparent cuticle that allows direct observation of internal structures under a dissecting microscope (Strange 2006; Tissenbaum 2012). With a short lifespan of approximately 12–17 days (maximum 25–30 days at 20 °C) (Gems and Riddle 2000), aging phenotypes appear within 7–10 days of hatching. The organism exhibits age-related declines similar to humans, including muscle atrophy, reduced mobility, immune decline, decreased stress resistance, and tissue degeneration (Tissenbaum 2012). Additional aging features include the accumulation of autofluorescent material, reduced pharyngeal pumping, and impaired neuronal transmission, leading to weakened mechanosensation, learning, and memory (Chew et al. 2013; Liu et al. 2013). These age-associated traits were used in our study to evaluate the health of *C. elegans* during aging and to compare treated and control groups. In all experiments, treatments were initiated in young adult worms to ensure that aging changes were measured in fully matured individuals with unperturbed development.

In our previous studies, both *C. chinensis* seed and *E. ulmoides* bark extracts exerted beneficial effects on *C. elegans* healthspan (Sayed et al. 2022, 2021). Treated worms exhibited extended lifespan and improved survival under heat and oxidative stress, as well as resistance to the pathogen *Photorhabdus luminescens*. In addition, both extracts reduced intracellular reactive oxygen species levels in 12-day-old adults. However, *C. chinensis* additionally enhanced locomotion, short-term memory, and mechanosensory responses while reducing intestinal autofluorescence in aged worms, indicating broader anti-aging benefits compared to *E. ulmoides* (Sayed et al. 2022, 2021). To explore whether these distinct physiological outcomes arise from different molecular mechanisms, we compared transcriptomic profiles of *C. elegans* treated with *E. ulmoides* and *C. chinensis* extracts. This approach provides deeper insight into how distinct TCM compounds modulate aging-related pathways in *C. elegans*.

## Methods

### Preparation of TCM extracts

*Eucommia ulmoides* bark and *Cuscuta chinensis* seeds were sourced from the Beijing Tong Ren Tang Chinese Medicine Company (Beijing, China). Organic extracts of these plants were prepared and provided by AnalytiCon Discovery GmbH (Potsdam, Germany). Briefly, 20 g of dried *C. chinensis* seeds or *E. ulmoides* bark were extracted at room temperature using a solvent mixture of tert-butyl methyl ether (MTBE), methanol (50:50, 75 mL total volume) followed by 100% methanol (volume 75 mL). Extraction was supported by 15 min of sonication and two hours of maceration. The extracts were combined, dried at 45 °C under a continuous airflow for four hours, and subsequently stored in the dark at 4 °C. Before experimental application, the extracts were freshly dissolved and diluted in DMSO.

### *Caenorhabditis elegans* maintenance

The wild-type N2 Bristol *C. elegans* strain and *Escherichia coli* feeding strain OP50 were obtained from the Caenorhabditis Genetics Center (Minneapolis, MN, USA). Worms were maintained on nematode growth medium (NGM) agar plates seeded with *E. coli* OP50, following the methods established by Sydney Brenner (1974). Synchronized worms were regularly generated by treating young adults with a 3% sodium hypochlorite solution until eggs were isolated, based on a protocol from Stiernagle (2006). The obtained eggs were incubated with overnight shaking in M9 buffer, and the hatched L1 larvae were transferred to new NGM plates the following day. Two days later, the nematodes reached the fourth larval stage (L4) and were transferred to the respective treatment plates.

### Treatment of *C. elegans*

*C. chinensis* and *E. ulmoides* extracts were dissolved in dimethyl sulfoxide (DMSO) to prepare stock solutions at a concentration of 60 mg/mL. These stock solutions were diluted to a final concentration of 30 μg/mL in both NGM agar plates and the corresponding bacterial food source. DMSO (0.05%) was used in the control feeding bacteria and NGM agar. Synchronized, untreated L4 larvae were transferred onto NGM agar plates supplemented with 2 mg/mL carbenicillin. 5-fluorodeoxyuridine (FUdR, 100 μM) was added to the NGM plates to inhibit progeny production (Hosono 1978). IPTG (1 mM) was only added to NGM plates that were used for RNAi experiments. The nematodes were incubated at 22 °C until they reached the adulthood stage required for each respective bioassay (described below).

### RNA-sequencing (RNA-seq) analysis

Total RNA was extracted from wild-type *C. elegans* that had been treated with either *E. ulmoides* or *C. chinensis* from the L4 larval stage until the 12th day of adulthood. Extraction was performed using the innuSPEED Tissue RNA Kit (Analytik Jena, Jena, Germany). On the 12th day of adulthood, approximately 3000–4000 worms were collected per tube, transferred into lysis tubes containing steel beads, and immediately stored at − 80 °C. RNA isolation followed the manufacturer’s protocol. To eliminate residual DNA, the TURBO DNA-free™ Kit (Thermo Fisher Scientific, Darmstadt, Germany) was applied as a final step. The RNA content of each sample was measured using a NanoDrop™ One Microvolume UV–Vis Spectrophotometer (Thermo Fisher Scientific, Waltham, USA), and RNA integrity was verified by gel electrophoresis.

RNA library preparation and transcriptome sequencing were conducted by Novogene Co., Ltd. (Beijing, China). Briefly, a total of 1 μg RNA per sample was used as input for RNA sample preparation. Sequencing libraries were generated using the NEBNext® Ultra™ RNA Library Prep Kit for Illumina® (NEB, USA), following the manufacturer’s recommendations. First-strand cDNA was synthesized using random hexamer primers and M-MuLV Reverse Transcriptase (RNase H −). Second-strand cDNA synthesis was subsequently performed using RNase H and DNA Polymerase I. To select cDNA fragments of approximately 150–200 bp, library fragments were purified using the AMPure XP system (Beckman Coulter, Beverly, USA).

PCR amplification was then performed using Phusion High-Fidelity DNA Polymerase, Universal PCR primers, and Index (X) Primer. Final PCR products were purified (AMPure XP system), and library quality was assessed using the Agilent Bioanalyzer 2100 system. Clustering of index-coded samples was performed on a cBot Cluster Generation System using the PE Cluster Kit cBot-HS (Illumina) according to the manufacturer’s instructions. After cluster generation, libraries were sequenced on an Illumina platform, and paired-end reads were generated.

The read count data were analyzed using DEBrowser (Kucukural et al. 2019), an R-based tool, in combination with the Bioconductor package edgeR. Differential expression analysis was performed following trimmed mean of M-values (TMM) normalization, and genes with counts per million (CPM) below one were filtered out. Genes were classified as differentially expressed (DEGs) if their *p*-value, adjusted via the Benjamini–Hochberg method in DEBrowser, was below 0.05 and their fold change exceeded 1.5 or was below 0.667. Further functional analysis of DEGs was conducted using the DAVID (Database for Annotation, Visualization, and Integrated Discovery; Huang et al. 2009a, 2009b) online platform to identify associated Kyoto Encyclopedia of Genes and Genomes (KEGG) pathways and Gene Ontology (GO) terms (Ashburner et al. 2000). The raw RNA-seq data have been submitted to NCBI’s Gene Expression Omnibus (GEO; Edgar et al. 2002) under the accession number GSE274478 (https://www.ncbi.nlm.nih.gov/geo/query/acc.cgi?acc=GSE274478).

### Quantitative polymerase chain reaction (qPCR)

Quantitative PCR (qPCR) analysis was performed using the Luna® Universal qPCR Kit (New England BioLabs, Frankfurt am Main, Germany) in conjunction with the MyiQ™ Single-Color RT-PCR Detection System (Bio-Rad, Hercules, CA, USA). Each sample was analyzed in three independent biological replicates, with technical triplicates for each biological replicate. Fold change calculations were performed using the method described by Pfaffl (2001), with *act-1* and *cdc-42* as reference genes for normalization. Detailed information on primer sequences, annealing temperatures, primer efficiencies, and the qPCR cycling protocol is provided in Supplementary Data S1 and S2.

### RNA interference

To downregulate the target gene via RNA interference (RNAi), the *far-3* RNAi feeding strain was constructed by restriction-based plasmid cloning (Supplementary Data S3). The target sequence from genomic *C. elegans* DNA was amplified using the *far-3* RNAi primer pair, which includes the restriction sites Pst-1 (forward) and Sac-II (reverse). The product (485 bp) was ligated into a L4440 plasmid (2790 bp) using T4 ligase. Before ligation, the PCR product and vector were purified using the Monarch® PCR & DNA Clean up Kit (5 μg). The DNA concentration and purity were determined via NanoDrop measurement. 50 ng of the vector was used in the ligation step while the mass of PCR product needed for the ligation was calculated to have the molar ratio 5:1 of vector to PCR product.

The plasmid was first transformed into *E. coli* DH5α cells (obtained from Thermo Fischer Scientific), then into *E. coli* HT115 (from the Caenorhabditis Genetics Center (Minneapolis, MN, USA)), and finally sequenced to verify the correct insert sequences. The BLASTN (Boratyn et al. 2012) results are shown in Supplementary Data S4.

Prior to use, the fresh feeding bacteria, which were grown at 37 °C overnight in LB medium spiked with 2 mg/mL carbenicillin, were treated with 1 mM IPTG for two hours, then washed with 3 g/L NaCl, and adjusted to a density of OD_595_ = 9. The bacteria were distributed on NGM plates containing 1 mM IPTG and 2 mg/mL carbenicillin at least one day before use. Nematodes fed with empty vector (EV) feeding bacteria served as a control.

### Verification of RNAi efficiency

To assess RNAi efficiency via bacterial feeding in *C. elegans*, single-worm RT-qPCR was conducted on the third day of adulthood, following the method described by Ly et al. (2015). At this developmental stage, four nematodes were rinsed in a drop of M9 buffer and placed into 4 µL of freshly prepared Worm Lysis Buffer in a PCR tube. Three biological replicates were prepared for nematodes exposed to *far-3* RNAi, and another three for the EV bacteria-treated group.

Lysis tubes were incubated in a thermocycler at 65 °C for 10 min, followed by 1 min at 85 °C to inactivate proteinase, and were then placed on ice. Genomic DNA removal and cDNA synthesis were performed using the Maxima H Minus cDNA Synthesis Kit (Thermo Fisher), according to the manufacturer’s instructions. To generate a negative control (− RT), 15 µL of each cDNA sample was divided into two equal portions; 7.5 µL were transferred into a separate PCR tube. For both the sample and control tubes, 7.5 µL of ddH₂O and 4 µL of 5 × RT buffer were added. The Maxima H Minus enzyme mix (1 µL) was added to the sample tubes, while 1 µL of ddH₂O was used for the controls. Samples were incubated using a predefined thermocycler protocol (see Supplementary Data S5). After incubation, 5 µL of ddH₂O was added to each tube, and samples were stored at − 80 °C until RT-qPCR analysis. The qPCR procedure was carried out as previously described.

### Lifespan and heat survival assays

To determine the effect of the downregulation of *far-3* on lifespan as well as survival after heat stress, 100–125 synchronized L4 larvae per treatment were transferred onto four small RNAi agar plates seeded with either the EV RNAi feeding bacteria (control), the *far-3* RNAi feeding strain, or the *far-3* RNAi feeding strain supplemented with 30 μg/mL *C. chinensis* extract. For the lifespan assay, the plates were incubated at 22 °C, and the worms were transferred to fresh treatment plates regularly every seven days. Living and dead nematodes were counted daily until all worms had died. Animals that had ruptured or left the agar surface were excluded from analysis.

For the heat stress assay, control and treated worms were incubated at 37 °C for 3 h on the 12th day of adulthood and the number of surviving nematodes was monitored daily to determine their heat stress resistance. The experiment was repeated independently twice.

### Swimming behavior

The swimming behavior of *far-3* RNAi-treated nematodes was assessed and analyzed using CeleST software (version 3.1) (Restif et al. 2014). On the 12th day of adulthood, 6–10 worms were placed into wells (0.5 mm depth, 10 mm diameter) on a microscope slide, filled with M9 buffer and covered with a coverslip to enhance visualization. Videos of 60 s were recorded for each well, with at least 50 nematodes analyzed per treatment group. For image processing, every other frame was extracted, and the grayscale and invert modes were applied using Adobe Photoshop (version 19.1.7). The wave initiation rate, activity index, brush stroke, and body wave number were subsequently quantified using CeleST.

### Autofluorescence

Autofluorescence in control and treated *C. elegans* was measured on the 12th day of adulthood, following the method described in Pincus et al. (2016). For each treatment group, approximately 20–50 nematodes were placed on a 2% agarose pad atop a microscope slide and immobilized using 1 M sodium azide. Red autofluorescence was visualized and recorded using an Axiolab fluorescence microscope equipped with a TRITC filter set (excitation: 546 nm; emission: 600 nm) and a ProgRes C12 digital camera. A 10 × objective was used to image the worms. Mean fluorescence intensities per nematode were quantified densitometrically using the CellProfiler software (McQuin et al. 2018).

### Statistical analysis

Statistical analysis was performed using a one-way ANOVA followed by Bonferroni’s multiple comparison test (https://astatsa.com/OneWay_Anova_with_TukeyHSD/) or a log-rank test via the Online Application for Survival Analysis (OASIS 2) (Han et al. 2016), with subsequent Bonferroni correction. Data are presented as mean ± SEM (standard error of the mean). Differences were considered statistically significant at adjusted *p*-values of *p* < 0.05 (*)*, p* < 0.01 (**), *p* < 0.001 (***), or *p* < 0.0001 (****).

## Results

### The transcriptome of *C. elegans* was strongly affected by treatment with *C. chinensis* extract

In a previous publication, we identified *C. chinensis* as a strong overall healthspan enhancer while the *E. ulmoides* extract only increased the stress survival of *C. elegans* (Sayed et al. 2021). We hypothesized that the transcriptomes mirror the phenotypic differences between the *C. chinensis* and *E. ulmoides* treatments and that the beneficial effects elicited by *C. chinensis* were due to the modulation of aging-related pathways.

To assess transcriptomic changes, RNA sequencing (RNA-seq) was conducted on both treated and untreated *C. elegans* on the 12th day of adulthood. Read count analysis was performed using the R-based tool DEBrowser (Kucukural et al. 2019), applying a fold change threshold of ≥ 1.5 and an adjusted *p*-value (*p*_adj_) ≤ 0.05. The RNA-seq expression profiles were further validated through RT-qPCR analysis (see Supplementary Data S6).

**Fig. 1:**
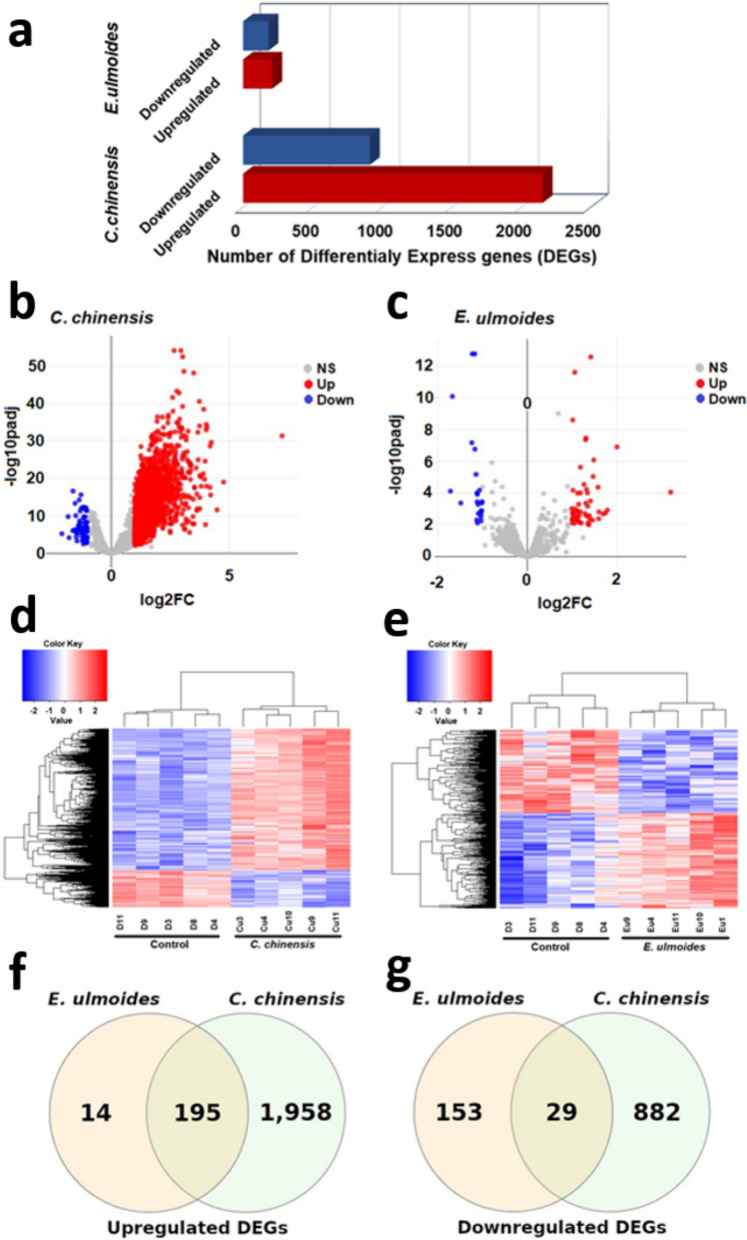
Quality control results of the RNA-seq read counts. **a** Transcriptional effect of *C. chinensis* and *E. ulmoides* on *C. elegans*. The volcano plots of **b** *C. chinensis* and **c** *E. ulmoides* show upregulated DEGs in red, downregulated DEGs in blue, and non-significant genes in grey. The heatmaps **(d, e)** represent the degree of changed gene expression by color-coding along a blue-red gradient; red indicates stronger expressed genes, and blue indicates lower expressed genes. Venn diagrams indicate the overlap of upregulated DEGs **(f)** and downregulated DEGs **(g)** after *C. chinensis* and *E. ulmoides* treatments

In nematodes exposed to *C. chinensis*, a total of 3064 differentially expressed genes (DEGs) were identified compared to the control group. Among these, 2153 genes were significantly upregulated, while 911 were downregulated (Fig. 1A and B; Supplementary Data S10). In contrast, *E. ulmoides* treatment resulted in the upregulation of 209 genes and downregulation of 182 genes (Fig. 1A and C; Supplementary Data S10). Clustering of gene expression profiles demonstrates a clear separation of all replicate experiments with treatment versus controls and further shows that the changes in expression patterns are highly distinct between *C. chinensis* and *E. ulmoides* treatments **(**Fig. 1D and E**).** Additionally, we compared the DEGs of the two treatments and found 195 genes up- and 29 genes downregulated in both treatments **(**Fig. 1F and G**)**. This means that 93% of the upregulated DEGs in the *E. ulmoides* treatment group were also found in the upregulated DEG list of *C. chinensis*-treated worms**.** These DEGs could be the basis for the common stress survival advantages in both treatment groups. On the other hand, only 9% of the upregulated DEGs in the *C. chinensis* group were also upregulated due to the *E. ulmoides* treatment. Thus, 1958 genes were uniquely upregulated in the *C. chinensis*-treated worms. Overall, the effect of the *C. chinensis* extract is much stronger and markedly different from that of the *E. ulmoides extract.*

### *Cuscuta chinensis* treatment enriched gene ontology terms associated with healthy aging

To obtain an overview of the particular functions of the DEGs, we performed a KEGG pathway and GO term analysis (Ashburner et al. 2000; Kanehisa et al. 2017). For all terms, a *p*-value < 0.05 and a fold change ≥ 1.5 were used to detect the significantly enriched terms. The complete lists of enriched terms are shown in Supplementary Data S11.

**Fig. 2:**
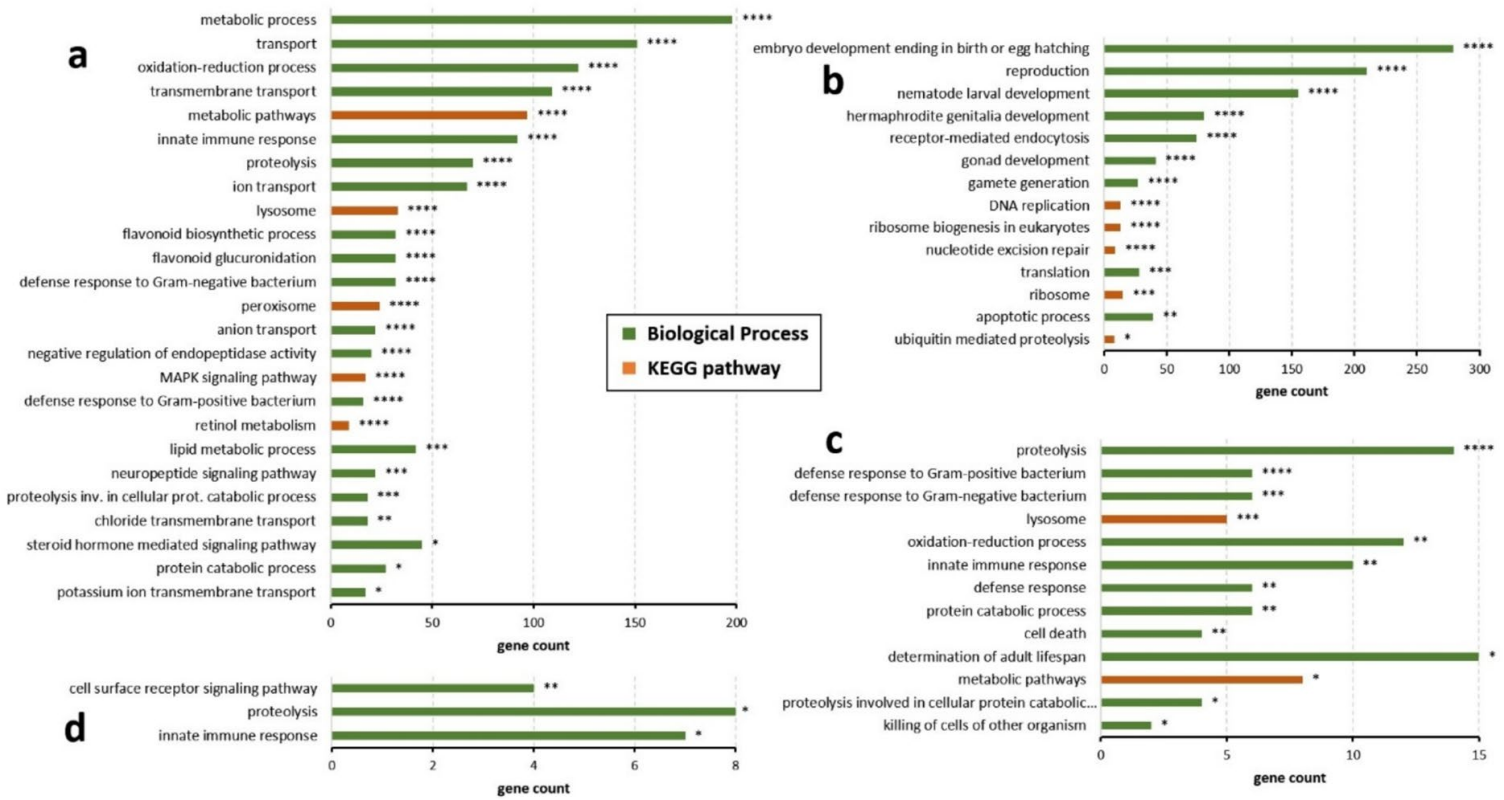
Gene Ontology and KEGG pathway analysis of upregulated and downregulated DEGs in treated *C. elegans*. Gene Ontology (GO) and KEGG pathway analysis results are displayed for upregulated (**a**) and downregulated (**b**) DEGs in *C. elegans* treated with *C. chinensis*, as well as upregulated (**c**) and downregulated (**d**) DEGs in *C. elegans* treated with *E. ulmoides*. Only GO terms belonging to the category “Biological Process” are displayed. The *p*-values are indicated by *(*p* < 0.05), ** (*p* < 0.01), *** (*p* < 0.001), or **** (*p* < 0.0001)

For the *C. chinensis* treatment group, KEGG pathways as well as gene ontology (GO) terms from the category “Biological Process (BP)” are shown in Fig. 2A and B. Several of the upregulated genes were associated with BP terms relevant to healthspan, such as “innate immune response” and “oxidation–reduction process” (Fig. 2A). KEGG pathway analysis indicated that many of the upregulated DEGs were involved in metabolic pathways, as well as in lysosomal and peroxisomal functions and retinol metabolism. Additionally, we observed that aged *C. elegans* treated with *C. chinensis* showed downregulation of several genes related to reproduction, including the BP terms “reproduction”, “nematode larval development”, “embryo development ending in birth or egg hatching”, and “hermaphrodite genitalia development” (Fig. 2B). Treatment of aged *C. elegans* with *E. ulmoides* extract resulted in a distinct enrichment pattern, with “proteolysis” and “defense response to Gram-positive bacterium” showing the most significant enrichment (Fig. 2C). In contrast, downregulated DEGs in the *E. ulmoides* group yielded only a few enriched GO terms, with limited statistical significance (Fig. 2D).

Based on the number and distribution of altered genes and pathways, *C. chinensis* induced a much broader spectrum of transcriptional changes compared to *E. ulmoides*. Overall, GO pathway analysis revealed that *C. chinensis* exerted a distinct influence on aging-related processes, characterized by the upregulation of immune-related genes and downregulation of genes linked to reproduction and development.

### *far-3* expression showed a 150-fold increase after treatment with *C. chinensis*

Next, we examined the genes that exhibited the most significant expression changes following *C. chinensis* treatment in *C. elegans*. While even moderately enriched DEGs may contribute to the healthspan effects of the extract, identifying such genes fell beyond the scope of this study. The ten most strongly up- and downregulated DEGs in aged *C. elegans* treated with *C. chinensis* are listed in Table 1. Among these, the most upregulated gene was *far-3*, with a log₂ fold change (log₂FC) of 7.2, corresponding to approximately an impressive 150-fold increase (validated by RT-qPCR, see Supplementary Data S6). This gene encodes a lipid- and retinol-binding protein specific to the nematode family. In addition, *C. chinensis* treatment significantly upregulated several cuticle collagen genes, including *col-143*, *col-122*, and *col-8* (log₂FC = 4.48, 4.20, and 4.03, respectively; see Tables 1 and 2). The most downregulated gene was *ins-23*, which is involved in the insulin signaling pathway and has hormonal activity. However, Table 1 also includes several genes with unknown functions, for which no link to healthy aging can be established at this stage.

**Table 1:** Top 10 enriched DEGs in aged *C. elegans* treated with *C. chinensis*

	Symbol	Description	Function and regulation	log_2_FC
Upregulated	*far-3*	Fatty Acid/Retinol binding protein	Lipid/Retinol binding activity	7.23
	ZK970.7	Similar to the *Onchocerca volvulus* Ov17 hypodermal antigen	Affected by stressors such as ionizing radiation and bacterial infection	4.75
	*col-143*	Collagen	A structural constituent of cuticle; Integral component of membrane	4.48
	*F48C1.9*	Enriched in hypodermis	Affected by *daf-16*, *daf-2*, and *glp-1*	4.24
	*col-8*	Collagen	Constituent of cuticle; Predicted to be part of collagen trimer	4.21
	*F37H8.5*	Ortholog of human IFI30 (IFI30 lysosomal thiol reductase)	Enables oxidoreductase activity	4.19
	*F23D12.11*	Enriched in excretory cell	Affected by *daf-16, daf-2, and skn-1*	4.18
	*T19B10.2*	Expressed in hypodermis	Mediates increased life span of *C. elegans*	4.06
	*col-122*	Collagen	Constituent of cuticle; Predicted to be part of collagen trimer	4.04
	*F55H12.4*	Expressed in anus, hypodermis, pharynx, and vulva		4.02
Downregulated	*ins-23*	Insulin related	Hormone activity	− 2.08
	*clec-266*	C-type lectin domain	Carbohydrate binding activity	− 1.81
	*F13E9.15*	Enriched in amphid sheath cell, hypodermis, and neurons	Affected by *daf-16*, *daf-2*, and *skn-1*	− 1.77
	*F59A7.7*	*–*	Cysteine synthase activity	− 1.61
	*fbxa-140*	F-box A Protein	Affected by *daf-12*, *elt-2*, and *sir-2.1*	− 1.61
	*F28A10.11*	*–*	Affected by *mrps-5*	− 1.54
	*his-60*	Histone	DNA binding activity and protein heterodimerization activity	− 1.52
	*his-73*	Histone	DNA binding activity and protein heterodimerization activity	− 1.49
	*aptf-4*	AP-2 Transcription factor family	DNA-binding transcription activator activity	− 1.48
	*T05D4.2*	Enriched in head mesodermal cell and neurons	Affected by daf*-16, daf-2*, and* eat-2*	− 1.47

Description and functions of the listed genes were based on Wormbase online database **(**Sternberg et al. 2024**)**

**Table 2:** Top 10 regulated gene families after *C. chinensis* and *E. ulmoides* extract treatment in aged *C. elegans*

*C. chinensis*					*E. ulmoides*				
Gene family	Gene name	Count			Gene family	Gene name	Count		
		Up	Down	Total			Up	Down	Total
*fbxa*	F-box A protein	22	25	222	*ttr*	Transthyretin-Related family domain	11	3	59
*nhr*	Nuclear hormone receptor family	45	1	283	*clec*	C-type Lectin	7	0	264
*ugt*	UDP-glucuronosyl-Transferase	32	0	67	*col*	Collagen	6	0	164
*ttr*	Transthyretin-Related family domain	30	0	59	*asp*	Aspartyl protease	6	0	18
*clec*	C-type Lectin	25	2	264	*fbxa*	F-box A protein	1	4	222
*col*	Collagen	17	1	164	*nspc*	Nematode specific peptide family, group C	4	0	20
*nlp*	Neuropeptide-Like Protein	17	0	74	*nlp*	Neuropeptide-Like Protein	3	0	74
*unc*	Uncoordinated	14	0	110	*fipr*	Fip (fungus-induced protein) related	3	0	29
*spp*	Saposin-like Protein family	11	0	31	*ugt*	UDP-Glucuronosyltransferase	3	0	67
*pgp*	P-Glycoprotein related	11	0	15	*cpr*	Cysteine protease related	3	0	8

The counts indicate the number of DEGs belonging to each affected gene family. The DEG count categories are, in order, upregulated (Up), downregulated (Down), and the total number of genes in each gene family (Total). All genes included in the ‘Up’- and ‘Down’-categories have an adjusted *p*-value (*p*_adj_) < 0.05 and a fold change > 1.5 or < –0.5

### No changes in lifespan and heat stress resistance after *C. chinensis*/*far-3 *RNAi or *far-3* RNAi treatment alone

Due to the strong upregulation of *far-3* in aged *C. elegans* treated with *C. chinensis*, we asked whether the overexpression of *far-3* was primarily responsible for the observed healthspan improvements in *C. chinensis*-treated nematodes found in Sayed et al. (2022; 2021)**.** We therefore downregulated *far-3* via RNAi in *C. elegans* and performed several bioassays. Additionally, we treated *far-3* RNAi *C. elegans* with *C. chinensis* to investigate whether the absence of *far-3* expression modifies the healthspan-enhancing effects of *C. chinensis*. The efficiency of the RNAi treatment was verified with a single-worm RT-qPCR, which showed that *far-3* was downregulated in nematodes treated with *far-3* RNAi alone, as well as in combination with the *C. chinensis* extract (Supplementary Data S7).

**Fig. 3:**
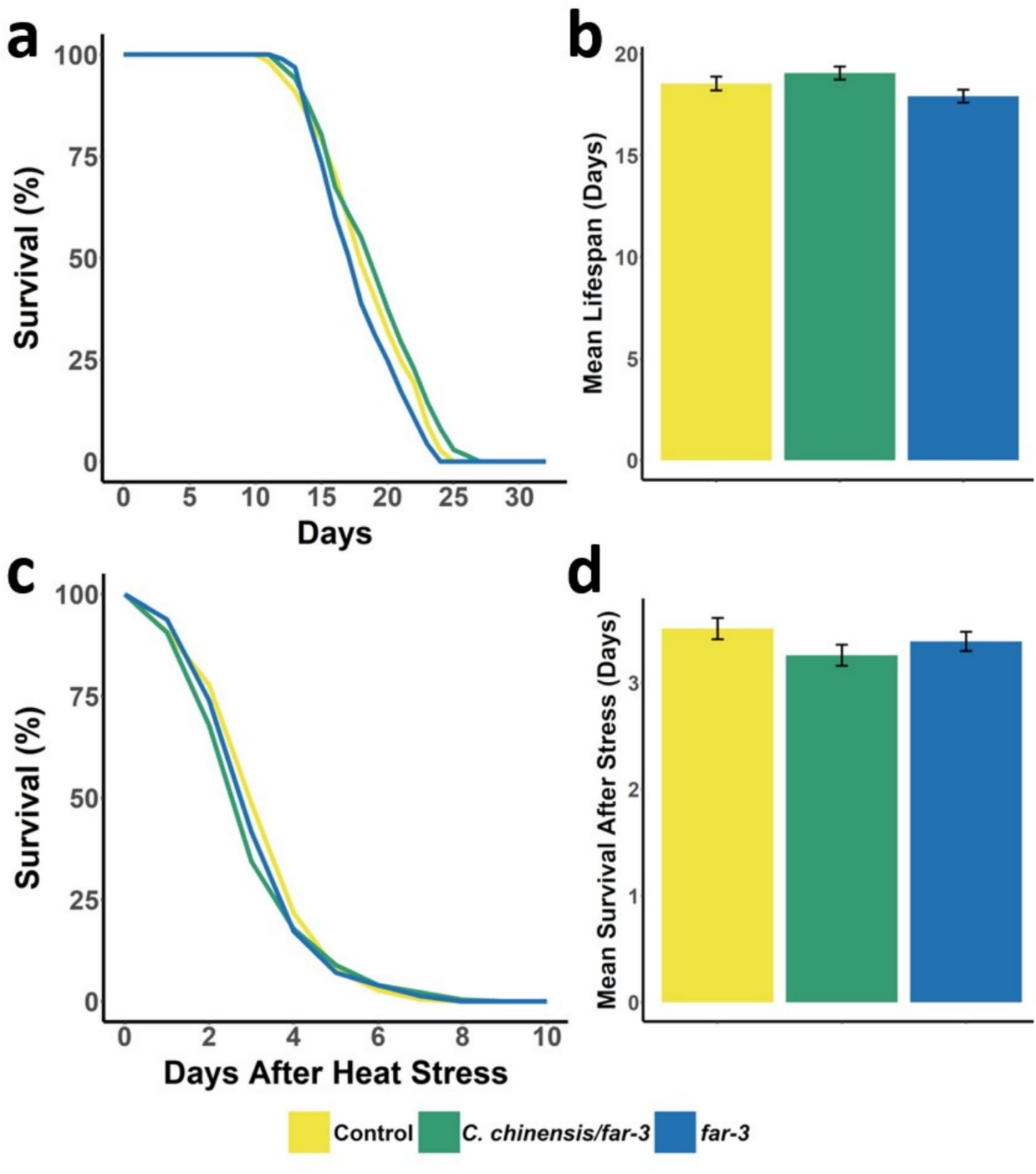
Lifespan and heat stress survival assays of *C. elegans* after treatment with 30 μg/mL *C. chinensis*/*far-3* RNAi or *far-3* RNAi alone. EV RNAi was used as a control. **a** Representative lifespan curves and resulting **b** mean lifespan ± SEM of each treatment (*n* > 90) are shown. **c** Worms were exposed to heat stress (3 h at 37 °C) on the 12th day of adulthood, and survival was monitored over the following days. The mean survival ± SEM is shown in (**d**). The significance of survival curves was determined using a log-rank test with post hoc Bonferroni correction. No significant differences were found (*p* < 0.05)

We previously reported that *C. chinensis* extract extends the mean lifespan of *C. elegans* by 24% (Sayed et al. 2021). To investigate the potential involvement of *far-3* in this effect, we assessed the lifespan of worms subjected to *far-3* RNAi, with or without additional *C. chinensis* treatment. Worms treated solely with *far-3* RNAi exhibited a mean lifespan of 17.92 ± 0.32 days, whereas those receiving both *far-3* RNAi and *C. chinensis* extract showed a significantly longer mean lifespan of 19.06 ± 0.32 days (Fig. 3A and B; *p*_adj_ = 0.0052). However, in comparison to the control group (18.54 ± 0.34 days), neither treatment led to a substantial change in mean lifespan, and no significant differences were observed in minimum, median, or maximum lifespan (see Supplementary Data S8).

Aging is associated with a diminished capacity to regulate the heat stress response, rendering older worms more susceptible to thermal damage. Based on previous findings that *C. chinensis* extract enhances heat stress resistance in *C. elegans* (Sayed et al. 2021), we examined whether *far-3* contributes to this effect. Worms treated with *far-3* RNAi, *far-3* RNAi plus *C. chinensis* extract, and untreated controls were exposed to a 3-h heat shock at 37 °C on day 12 of adulthood. Analysis of survival curves (Fig. 3C) and mean survival times (Fig. 3D, Supplementary Data S9) revealed no significant differences in heat stress resistance between the groups. The mean survival durations were 3.51 ± 0.10 days for the control group, 3.26 ± 0.10 days for the *C. chinensis*/*far-3* RNAi group, and 3.39 ± 0.09 days for the *far-3* RNAi-only group.

Although *far-3* does not appear to directly impact lifespan or heat stress resistance in untreated worms, *C. chinensis*-treated worms lacking *far-3* failed to exhibit the previously observed benefits of increased lifespan and heat tolerance. This indicates that *far-3* is crucial for mediating or supporting the protective effects of *C. chinensis*.

### *Cuscuta chinensis* in combination with *far-3* RNAi decreased locomotor fitness

Next, we investigated the effect of *far-3* knockdown, with and without *C. chinensis* extract treatment, on the swimming behavior of *C. elegans* on day 12 of adulthood by tracking and analyzing their movement in liquid medium. The measured parameters—wave initiation rate, body wave number, activity index, and brush stroke—characterize locomotor fitness across treatment and control groups. With advancing age, a decline in wave initiation rate, activity index, and brush stroke is typically observed, whereas body wave number increases. We previously showed that *C. chinensis* exposure leads to improved swim performance in all measured parameters on the 12th day of adulthood (Sayed et al. 2021).

**Fig. 4:**
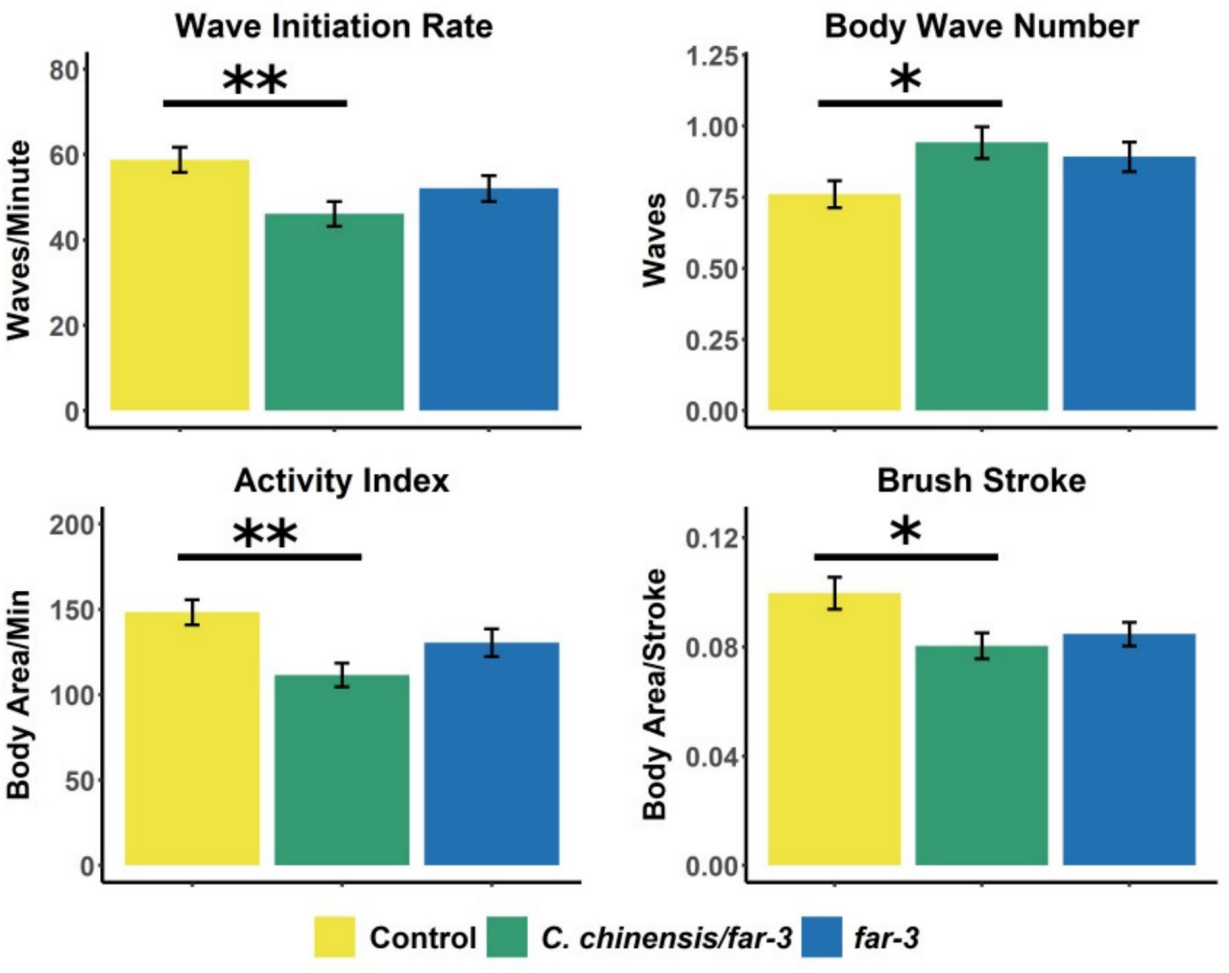
Swimming bioassay of *C. elegans* after treatment with 30 μg/mL *C. chinensis*/*far-3* RNAi or *far-3* RNAi alone. EV RNAi was used as a control. Four measures scoring swim behavior (wave initiation rate, body wave number, activity index, and brush stroke) were calculated based on tracking results of individual worms. Results are shown as the mean of *n* ≥ 80 individuals per treatment, with error bars indicating SEM. Statistical significance was determined with a one-way ANOVA and post hoc Bonferroni test; *p*_Bonf_ < 0.05, *p*_Bonf_ < 0.01, *p*_Bonf_ < 0.001, and *p*_Bonf_ < 0.0001 are indicated with *, **, ***, and ****, respectively

*far-3* RNAi worms treated with *C. chinensis* exhibited a significant reduction in locomotor abilities compared to the control group: the wave initiation rate, activity index, and brush stroke decreased by 21.5%, 25%, and 19.3%, respectively, while the body wave number increased by 23.8% (Fig. 4). Although *far-3* RNAi-treated *C. elegans* (without *C. chinensis* extract) showed a similar trend, the changes were not statistically significant (*p*_*adj*_ > 0.05). These results indicate that the TCM extract requires the expression of *far-3* to induce an improvement in locomotion.

### Knockdown of *far-3* increased accumulation of red autofluorescent material

Red autofluorescence in *C. elegans* (excitation/emission: 546/600 nm) is a widely used healthspan marker that increases progressively with age (Pincus et al. 2016). Although the exact composition of this primarily intestinal autofluorescent buildup remains unclear, previous studies suggest it includes compounds such as lipofuscin, advanced glycation end products, flavins, and collagen (Knight and Billinton 2001; Tissenbaum 2012).

**Fig. 5:**
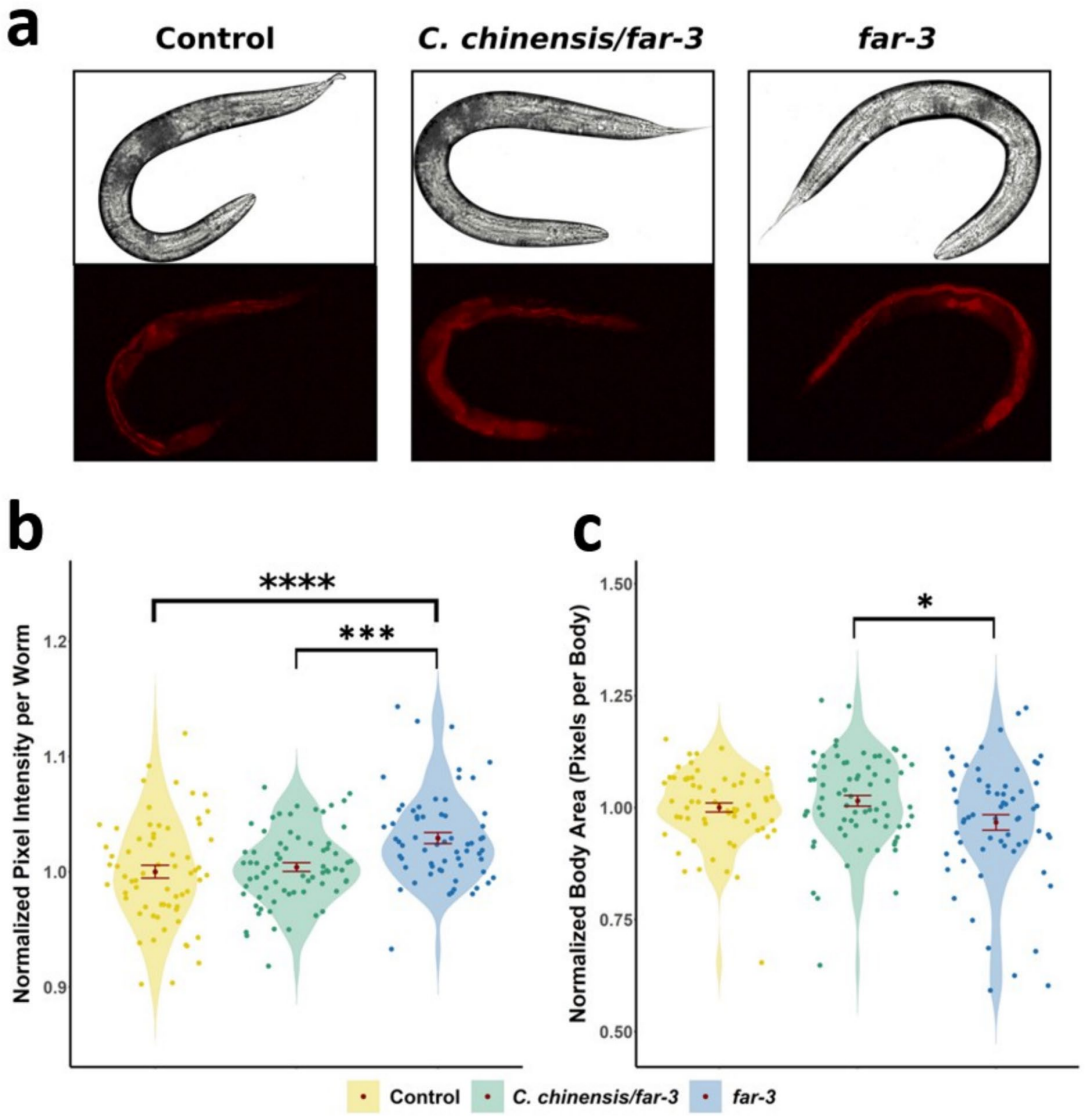
Red autofluorescence accumulation and body size assay of *C. elegans* treated with either *far-3* RNAi plus 30 μg/mL *C. chinensis* extract or *far-3* RNAi alone. Empty vector (EV) was used as a control. **a** Representative bright-field and red fluorescence images of the control and treated *C. elegans* nematodes on the 12th day of adulthood. **b** Red autofluorescence of individual nematodes (*n* ≥ 60) normalized to the body size and to the mean autofluorescence of the control EV-treated nematodes. Measurements were quantified densitometrically. **c** Body size of individual nematodes was calculated from the number of pixels per body (*n* ≥ 60) and normalized to the mean size of the control. Both violin plots show the distribution of measurements for individual nematodes, represented by grey dots. The red dot and error bars represent the mean ± standard error of the mean (SEM). Statistical significance was determined with a one-way ANOVA and post hoc Bonferroni test; *p*_Bonf_ < 0.05, *p*_Bonf_ < 0.01, *p*_Bonf_ < 0.001, and *p*_Bonf_ < 0.0001 are indicated with *, **, ***, and ****, respectively

On day 12 of adulthood, *far-3* RNAi-treated worms exhibited a 2.92% increase in red autofluorescence compared to control animals (*p*_adj_ = 0.000091), whereas this increase was absent in *far-3* RNAi worms additionally treated with the *C. chinensis* extract (Fig. 5B). Interestingly, treatment with *C. chinensis* led to a 2.52% reduction in autofluorescence in *far-3* RNAi worms compared to the untreated *far-3* knockdown group (*p*_adj_ = 0.00033). These results suggest that silencing *far-3* promotes the accumulation of age-related autofluorescent material—an effect that is mitigated by *C. chinensis* supplementation. Treatment with *C. chinensis* has been shown to lessen the buildup of red autofluorescent material in aged *C. elegans* (Sayed et al. 2021), a marker associated with better physiological condition and delayed aging. However, this effect appears to depend on the presence of FAR-3, as autofluorescence levels in the *C. chinensis*/*far-3* RNAi group remained comparable to those in the untreated control group (Fig. 5B).

To assess potential differences in body size, we also measured the body area of each worm (in pixels per individual). Since fully developed adult hermaphrodites consist of 959 somatic cells, variations in body area could reflect changes in cell size. However, neither treatment resulted in a significant difference in body area compared to the control group (Fig. 5C). Notably, *far-3* RNAi nematodes displayed a broader distribution in body size relative to those treated with both *far-3* RNAi and *C. chinensis* extract, which was also reflected in the higher SEM values (SEM_*far-3*_ = 0.0172; SEM_*C. chinensis/far-3*_ = 0.0119). Interestingly, worms treated with *far-3* RNAi alone exhibited a 4.8% reduction in body area (*p*_adj_ = 0.033) compared to those treated with both *far-3* RNAi and *C. chinensis*. Thus, neither the *C. chinensis* treatment in presence (Sayed et al. 2021) nor in absence of FAR-3 affected body size. However, *far-3* RNAi alone caused a slight reduction in worm size.

## Discussion

Aging is a multifaceted biological process characterized by a gradual decline in physiological functions, increased susceptibility to chronic diseases, and ultimately death (de Magalhães 2025). Our previous work demonstrated that *E. ulmoides* bark and *C. chinensis* seed extracts both extend the healthspan of *C. elegans*, although they elicited different phenotypic outcomes (Sayed et al. 2022, 2021). *E. ulmoides* primarily enhanced stress resistance and lifespan, whereas *C. chinensis* acted as a broader healthspan promoter, improving not only stress resistance and lifespan but also locomotion, pharyngeal pumping, and cognitive performance. To elucidate the underlying mechanisms, we analyzed the transcriptional profiles of nematodes treated with either extract. We identified overlapping gene expression patterns that may account for the shared effects on stress resistance and lifespan, as well as *C. chinensis*-specific expression signatures potentially responsible for its additional health-promoting effects. This comparative analysis provides insight into the molecular mechanisms driving the healthspan-enhancing activities of these two extracts.

### Shared induction of genes involved in immune defense, lysosomal function and protein homeostasis could underlie enhanced stress resistance and longevity in *C. chinensis-* and *E. ulmoides*-treated *C. elegans.*

One key question that RNA-seq analysis can address is whether the two plant extracts enhance stress resistance through similar changes in gene expression. Following treatment with *C. chinensis* and *E. ulmoides*, 195 genes were found to be upregulated in both groups, whereas only 29 genes showed shared downregulation. Interestingly, both extracts modulate the expression of genes associated with cellular maintenance and stress adaptation, such as immune defense, lysosomal activity, and proteolysis. Lysosomes are essential organelles that mediate the degradation and recycling of cellular components and play a crucial role in lifespan extension (Carmona-Gutierrez et al. 2016; Sun et al. 2020) and in the elimination of pathogenic organisms (Ballabio and Bonifacino 2020; de Magalhães 2025; Mullins and Bonifacino 2001; Pu and Qi 2024; Saftig and Puertollano 2021).

One of the most prominently activated gene families in both extract-treated groups was the lectin family, which is widely recognized for its role in immune responses across various organisms (Pees et al. 2021). Both extracts also upregulated the expression of *hsp-70*, which encodes a heat shock protein, and *mtl-1*, one of the two metallothioneins in *C. elegans* (Supplementary Data S10). These gene families are well known for their roles in promoting stress resistance and longevity in the nematode (Murshid et al. 2013; Swindell 2011). Furthermore, a notable increase in the expression of proteolysis-related genes was observed in aged worms treated with *C. chinensis* (70 genes) and *E. ulmoides* (14 genes), suggesting enhanced protein turnover and improved cellular homeostasis. Proteolysis, the regulated degradation of damaged or misfolded proteins, is crucial for maintaining proteostasis, which declines with age, leading to the accumulation of dysfunctional proteins and a deterioration in physiological function (Hamazaki and Murata 2024; Rai et al. 2022). In *C. elegans*, proteolysis has been shown to enhance stress resistance, confer protection against proteotoxic challenges in multiple tissues, and extend lifespan (Anderson et al. 2022; Panagiotidou et al. 2023).

In summary, the modulation of genes associated with stress response, immune defense, lysosomal function, and proteolysis could be the common underlying molecular cause for improved stress resistance and longevity in *C. chinensis-* and *E. ulmoides*-treated nematodes. This broad range of transcriptional responses is consistent with the multi-target nature described for several natural extracts (Herranz-López et al. 2018; Koeberle and Werz 2014; Namdeo et al. 2020; Vrabec et al. 2023) and may reflect the synergistic interactions often observed in complex phytochemical mixtures (Wagner and Ulrich-Merzenich 2009; Williamson 2001).

### Potential involvement of muscle-, collagen-, and neuron-associated genes in the effects of *C. chinensis* on locomotion and mechanosensation

Motor ability is a key indicator of organismal health, with age-related declines in muscle mass (sarcopenia) and strength (dynapenia) leading to impaired movement (Baumann et al. 2016; Jonk et al. 2025). Treatment of *C. elegans* with *C. chinensis* seed extract until day 12 of adulthood resulted in improved locomotion (Sayed et al. 2021) and the upregulation of genes associated with muscle integrity and cuticle maintenance. One of the most enriched gene ontology (GO) terms identified was “striated muscle-dense body” (Supplementary Data S11). In addition, the expression of 14 *unc* (uncoordinated) genes was found to be increased, which are known to affect movement abilities in *C. elegans* (Moerman et al. 1988). In parallel, the upregulation of 17 collagen genes indicates enhanced stability of the cuticular framework supporting locomotion (Johnstone 1994; Shin et al. 2011).

*Cuscuta chinensis* treatment also improved mechanosensation and short-term memory in aged worms (Sayed et al. 2022). Transcriptomic data revealed increased expression of several *mec* genes (*mec-5, mec-7, mec-12,* and *mec-17*) (Supplementary Data S10), which encode proteins crucial for touch-receptor neuron function and microtubule stability (Chalfie and Thomson 1982; Goodman and Sengupta 2019; Krieg et al. 2015; Neumann and Hilliard 2014). Upregulation of *unc-43* and *kin-2*, both involved in CaMKII and cAMP signaling, may underlie improved associative memory (Stein and Murphy 2014), while differential expression of proteolysis-related genes suggests a contribution of the ubiquitin–proteasome system to synaptic plasticity (Farrell et al. 2023; Patrick et al. 2023).

Together, these findings suggest that *C. chinensis* enhances muscular and neuronal performance in *C. elegans* through coordinated regulation of collagen, *unc*, and *mec* genes, thereby improving locomotion and sensory function. The simultaneous upregulation of *unc-43* and *kin-2*, along with proteolysis-related genes of the ubiquitin–proteasome pathway, may further support memory formation and counteract neurodegenerative decline.

### *far-3* could play an important role in the *C. chinensis*-induced healthspan improvement

Transcriptomic analysis of *C. elegans* treated with *C. chinensis* revealed *far-3* as the most strongly upregulated gene, exhibiting a ~ 150-fold increase in expression. Although large expression changes are not proof of functional involvement, such exceptional upregulation warrants further investigation. FAR proteins (Fatty Acid and Retinol-binding proteins) are nematode-specific and represent a substantial part of the excreted/secreted proteome, playing roles in lipid and retinol absorption and transport (Garofalo et al. 2003b). While analogous lipid- and retinoid-binding proteins in mammals predominantly adopt β-sheet structures, *C. elegans* FAR proteins display conserved α-helical folds with distinct hydrophobic pockets, allowing selective fatty acid and retinol binding (Garofalo et al. 2003a, 2003b). Functionally, *far-3* expression has been associated with immune responses, osmotic stress, and intercellular fatty acid transport (Burton et al. 2018; Erkut et al. 2016; Pujol et al. 2008). Moreover, FAR-3 was identified among a group of 14 proteins that increased with age in long-lived *daf-2(e1370)* mutants but not in wild-type worms (Narayan et al. 2016).

Silencing *far-3* led to increased red autofluorescence, suggesting that reduced FAR-3 expression may accelerate aging. Interestingly, worms treated with both *far-3* RNAi and *C. chinensis* extract until day 12 of adulthood did not show significant changes in autofluorescence compared to controls. This suggests that in the absence of FAR-3, *C. chinensis* may be unable to trigger the mechanisms that reduce autofluorescence and enhance healthspan. However, *C. chinensis* treatment still partially mitigated the autofluorescence increase induced by *far-3* RNAi, implying that its healthspan-promoting effects may also involve FAR-3-independent pathways. Thus, while FAR-3 seems to play a role in modulating autofluorescence, the healthspan-promoting effects of *C. chinensis* are likely mediated by a combination of FAR-3-dependent and -independent pathways.

FAR-3 may also contribute to improved stress resistance in *C. chinensis*-treated *C. elegans*. Although heat stress does not induce *far-3* expression in wild-type worms (Jovic et al. 2017; Lee et al. 2015), the thermotolerance-enhancing effect of *C. chinensis* in aged wild-type worms (Sayed et al. 2021) was abolished by *far-3* knockdown. Thus, *far-3* is likely not directly involved in the canonical heat shock response in *C. elegans* but may be essential for mediating the thermotolerance effect of *C. chinensis*. The role of FAR-3 in thermotolerance may be associated with its function as a fatty acid and retinol-binding protein. Although few connections between retinoids and heat tolerance are currently known, some studies have begun to explore this relationship (Maya-Soriano et al. 2013a, 2013b; Yue et al. 2022; Zhu et al. 2019). Fatty acids, however, have a well-established link to thermotolerance, particularly through homeoviscous adaptation: the ability of cells to modify their membrane structure to maintain proper fluidity in response to temperature changes (Ernst et al. 2016). Indeed, transcriptomic data revealed upregulation of several fatty acid metabolism genes, including elongases, desaturases, and *fasn-1* (Supplementary Data S10).

Furthermore, locomotion assays suggest a role for FAR-3 in muscle integrity. Worms subjected to *far-3* RNAi tended to exhibit reduced motor function, while the additional treatment with *C. chinensis* extract caused a marked decline in all four measured locomotion parameters compared to untreated controls. This indicates that FAR-3 is required for the locomotion-enhancing effects of *C. chinensis*. Multiple studies have highlighted the roles of fatty acids and retinoids in regulating muscle cell function and locomotion (Berraaouan et al. 2025; El Haddad et al. 2017; Fang et al. 2016; Gao et al. 2015; Zhang et al. 2023). Fatty acids are essential for protein myristoylation, endoplasmic reticulum (ER) homeostasis, and movement (Tang et al. 2021) and disruption of fatty acid biosynthesis impairs myosin organization in body-wall muscle cells, leading to mobility defects in *C. elegans* (Tang et al. 2021). Retinoids have been widely studied for their role in muscle integrity and regeneration (Di Rocco et al. 2015; Fraczek et al. 2025; Gudas 2022; Samara et al. 2024). In mice, all-trans retinoic acid increased the capacity for cellular fatty acid oxidation in skeletal muscles (Amengual et al. 2008; Shirasawa et al. 2021). Furthermore, supplementation with retinoic acid receptor agonists in mice decreased adipogenic differentiation in skeletal muscle by muscle fatty infiltration, a process associated with age (Shirasawa et al. 2021). Interestingly, RNA-seq analysis of *C. elegans* treated with *C. chinensis* revealed significant upregulation of the KEGG pathway “retinol metabolism”.

Taken together, our findings suggest that FAR-3 may play a central role in mediating the healthspan-promoting effects of *C. chinensis* in aged worms, linking lipid and retinoid metabolism with stress responses and muscle maintenance. However, it is important to note that these results are preliminary and do not provide direct evidence that FAR-3 affects healthspan-related phenotypes through lipid metabolism or retinol signaling. Nonetheless, FAR-3 represents a promising candidate for further investigation into the molecular mechanisms underlying the healthspan benefits of *C. chinensis*.

### *Cuscuta chinensis* modulates genes implicated in antagonistic pleiotropy and insulin-like signaling

The insulin/IGF-1 signaling (IIS) pathway is one of the key regulators of metabolism, stress resistance, and lifespan in *C. elegans* (Berk 2024). When the insulin-like receptor DAF-2 is active, it suppresses the FOXO transcription factor DAF-16, favoring growth and reproduction. In contrast, when IIS activity is reduced, DAF-16 translocates to the nucleus and activates genes that promote stress resistance and longevity (Ham et al. 2024; Sun et al. 2017).

Treatment with *C. chinensis* downregulated a distinct subset of insulin-like peptide (ILP) genes (*ins-10*, *ins-20*, *ins-22*, *ins-23*, *ins-37*), with *ins-23* showing the strongest decrease, and concurrently upregulated *ins-7*. In *C. elegans*, ILPs form a transcriptionally interlinked regulatory network that controls DAF-2/DAF-16 signaling and thereby specifies physiological outcomes (Fernandes de Abreu et al. 2014). Reduced expression of agonistic ILPs or an altered balance between agonists and antagonists is expected to diminish DAF-2 activity, promote DAF-16 nuclear translocation, and activate stress-protective genes, consistent with the enhanced stress resistance and extended lifespan observed after *C. chinensis* treatment. Although INS-23 and INS-37 have been proposed to function as DAF-2 antagonists, their downregulation, together with that of multiple agonistic ILPs (*ins-10*, *ins-20*, *ins-22*), likely reflects compensatory transcriptional crosstalk within the ILP network. Such coordinated regulation can produce a net reduction in insulin/IGF signaling, as seen in *ins-23* mutants that also exhibit increased longevity and stress tolerance (Fernandes de Abreu et al. 2014). Thus, the combined transcriptional adjustments of ILPs following *C. chinensis* exposure probably shift the network toward a low-IIS state and contribute to the extended healthspan.

Furthermore, *C. chinensis* treatment resulted in downregulation of several genes involved in reproduction and germline development. Although *C. chinensis* slightly delayed reproduction in *C. elegans*, total offspring numbers were unchanged (Sayed et al. 2021). Notably, RNA-seq data showing downregulation of reproduction- and development-related genes were obtained from aged, post-reproductive nematodes, raising the question of how such late-life gene regulation might influence health and survival. In *C. elegans*, there is strong evidence that the downregulation or removal of reproductive functions in adulthood can enhance somatic health and extend lifespan. Classic experiments showed that removal of germline cells extends lifespan, suggesting that reproductive tissues influence somatic aging (Hsin and Kenyon 1999). This longevity extension requires activation of conserved signaling pathways, notably the transcription factor DAF-16 and lipid-derived hormonal cues that reprogram intestinal metabolism and enhance stress resistance (Antebi 2013). Importantly, post-reproductive RNAi screens have revealed that late-life knockdown of reproduction-related genes can improve healthspan and lifespan, suggesting that suppression of reproduction pathways during aging exerts direct beneficial effects (Wilhelm et al. 2017). This is in line with antagonistic pleiotropy: genes that promote early reproduction can reduce longevity, while their downregulation later in life alleviates this trade-off in favor of somatic maintenance (Wu et al. 2022a). Comparable phenomena in *Drosophila*, where germline ablation modulates insulin signaling and extends lifespan, indicate that reproductive signaling and its influence on aging are evolutionarily conserved (Flatt et al. 2008). Collectively, these findings suggest that downregulation of reproductive gene activity in later life contributes causally to improved healthspan and longevity across taxa.

Interestingly, both *ins-23* and *far-3* are involved, directly or indirectly, in controlling lipid metabolism and stress protection. While INS-23 acts upstream as a hormonal signal regulating IIS and energy balance, FAR-3 functions downstream by binding fatty acids and retinoids to support membrane and cellular health. Their coordinated changes after *C. chinensis* treatment may indicate a broader adjustment of endocrine and metabolic pathways that together promote stress resistance and longevity.

### Comparative transcriptomic interpretation of *C. chinensis* and *E. ulmoides* extract treatment

Both *C. chinensis* and *E. ulmoides* extracts enhanced stress resistance and lifespan in *C. elegans*. RNA-seq analysis showed that both treatments activated conserved effector pathways involved in lysosomal biogenesis, proteolysis, and innate immunity, key processes supporting cellular maintenance and detoxification. These shared signatures indicate convergence on a common stress-resilience network that sustains proteostasis and counteracts age-related decline.

Despite this overlap, *C. chinensis* exhibited a broader transcriptomic remodeling, characterized by activation of several upstream regulators. Only *C. chinensis* treatment resulted in upregulation of *hlh-30*, a master regulator of lysosomal and autophagic genes (Lapierre et al. 2013; Zhong and Richardson 2024), and *hif-1*, a hypoxia-inducible transcription factor integrating metabolic and stress signals to modulate longevity (Belapurkar et al. 2023; Feng et al. 2024), together with modulation of several insulin-like peptides that regulate the IIS network (Matsunaga et al. 2018; Nele 2021). These transcriptional patterns suggest that *C. chinensis* modulates the aging process through higher-level regulatory nodes that coordinate stress defense, energy metabolism, and reproduction, whereas *E. ulmoides* primarily reinforces the downstream execution of these processes.

Interestingly, *E. ulmoides* exposure modulated fewer than 400 genes, whereas *C. chinensis* altered over 3000. Such a strong transcriptional response aligns well with the tendency of complex natural extracts to trigger extensive transcriptomic reprogramming in *C. elegans,* frequently encompassing several hundred to thousands of differentially expressed genes (Ding et al. 2024; Kumarasingha et al. 2019; Miao et al. 2025; Spiegler et al. 2017). This broad response likely arises from the chemical diversity of natural extracts, which contain numerous bioactive metabolites capable of simultaneously engaging multiple cellular stress and metabolic pathways. Indeed, the phytochemical complexity of both extracts, including diverse secondary metabolites and quantitative differences between them, has been demonstrated by UPLC-MS/MS profiling (Sayed et al. 2022).

Mild xenobiotic and redox stress can activate conserved stress-response transcription factors such as SKN-1, DAF-16, and HSF-1, as well as members of the large nuclear hormone receptor (NHR) family (Brunquell et al. 2016; Jones et al. 2013; Tullet 2015; Turner et al. 2024). Because each of these regulators controls extensive downstream gene networks involved in detoxification, proteostasis, and lipid metabolism, their simultaneous activation produces a large and coordinated transcriptional shift. Such global remodeling is consistent with hormesis, a phenomenon in which a mild stress stimulus enhances cellular defense capacity and stress resistance, as previously described for several natural extracts (Calabrese et al. 2019; Caruso et al. 2024; Ding et al. 2024; Herranz-López et al. 2018; Miao et al. 2025; Skaperda et al. 2021) and also proposed for *C. chinens*is (Sayed et al. 2021). The pronounced upregulation of *far-3*, encoding a fatty-acid and retinol-binding protein, further suggests that lipid mobilization and transport processes play a central role in this adaptive response, as similar lipid-binding proteins have been implicated in stress adaptation and metabolic remodeling in *C. elegans* (Kumarasingha et al. 2019; Spiegler et al. 2017).

Collectively, these findings support a model in which both plant extracts enhance stress resilience through conserved longevity pathways, yet *C. chinensis* engages additional upstream transcriptional and hormonal layers that broaden its physiological benefits and explain the large numbers of DEGs.

### Conclusions, limitations and future directions

This study provides new molecular insight into how *C. chinensis* and *E. ulmoides* promote healthspan in *C. elegans*. Both extracts enhanced stress resistance and lifespan, likely via shared activation of immune defense, lysosomal, and proteolytic pathways that support cellular maintenance. However, only *C. chinensis* additionally improved locomotion, short-term memory, mechanosensory response, and reduced intestinal autofluorescence in aged worms, indicating broader anti-aging effects. Consistent with the broad spectrum of observed phenotypes, *C. chinensis* induced extensive transcriptomic remodeling with over 3000 differentially expressed genes. The modulation of collagen-, *unc*-, and muscle-associated genes may explain improved locomotion, while upregulation of *mec* genes could underlie enhanced mechanosensation. Furthermore, *far-3*, encoding a fatty acid and retinol-binding protein and the most strongly induced gene, may play a central role in healthspan enhancement during *C. chinensis* treatment. Additionally, *C. chinensis* influenced genes linked to antagonistic pleiotropy and insulin-like signaling, suggesting broader engagement of aging-related pathways. Together, these findings point to hormesis-driven, systemic reprogramming as a likely mechanism underlying the health-promoting effects of *C. chinensis*.

Although this study provides valuable insights into the mechanisms of *C. chinensis* and *E. ulmoides*, several limitations should be noted. The transcriptomic data represent only an initial step, as functional validation at the protein and metabolic levels remains outstanding. Protein abundance and activity often better reflect biological outcomes than mRNA levels (Evans 2015), thus the functional relevance of key genes such as *far-3* and *ins-23* has yet to be confirmed. By using overexpression strains, GFP-reporter assays, proteomics, and lipid profiling, future studies should ultimately determine whether FAR-3 and insulin-like signaling mediate the observed benefits on healthspan and lifespan, and further elucidate the underlying mechanisms, temporal dynamics, and tissue-specific contributions governing this regulation. In addition, the use of fluorescent reporter strains, enzymatic assays, immunodeficient mutants, and lysosomal activity assays will enable monitoring of autophagy and protease activity in extract-treated nematodes, as well as the role of the immune system in the healthspan-promoting effects, thereby clarifying their contribution to the observed outcomes. Undoubtedly, it would also be interesting to determine whether the downregulation of reproduction- and development-related genes occurs as well in younger nematodes during the reproductive phase. Ultimately, extending these analyses to mammalian or cell-culture models will be necessary to test whether the mechanisms uncovered in *C. elegans* are conserved and to evaluate their translational relevance for healthy aging.

In addition, several inherent limitations of transcriptomics itself should be considered. According to Cui et al. (2021), high variation in transcript expression can strongly affect the reproducibility of RNA-seq differential expression results. To mitigate such variability, the authors recommend increasing the number of biological replicates and applying appropriate normalization and filtering procedures rather than relying solely on harsher statistical cutoffs. In line with these recommendations, our analysis employed TMM normalization, excluded low-abundance genes, and used five biological replicates with each 3000–4000 nematodes per condition. Differential expression was determined using Benjamini–Hochberg adjusted *p*-values (< 0.05) and a fold-change cutoff of ± 1.5. In total, our procedure provides a robust and reproducible framework consistent with the best practices highlighted by Cui et al. (2021).

Furthermore, Dam et al. (2023) showed that alternative splicing is a major regulator of human biology and suggested that many RNA-seq studies may overlook critical biological insights by ignoring isoform-level regulation. Alternative splicing is less common in *C. elegans* (Ramani et al. 2011); however, several genes, particularly from the *unc* family and other key players of the nervous and muscular system (Tan and Fraser 2017; Weinreb et al. 2025) generate multiple isoforms through alternative splicing. Isoform-resolved or long-read sequencing could help capture these gene-specific events more accurately.

Importantly, large fold changes in gene expression do not automatically imply functional relevance (Evans 2015; Rodriguez-Esteban and Jiang 2017), suggesting that the biological significance of the top 10 enriched DEGs shown in Table 1 should be interpreted with caution. Nevertheless, several studies indicate that pronounced transcriptional shifts can represent meaningful biological signals when interpreted carefully. Li et al. (2022) cautioned that standard RNA-seq pipelines may inflate false positives, emphasizing the importance of stringent statistical control and reproducibility. Liu et al. (2023) found that highly expressed genes are consistently recovered across RNA-seq platforms, suggesting that these DEGs often reflect robust biological changes. Similarly, Carbonetto et al. (2023) showed that strong expression changes often involve co-regulated genes within specific pathways, suggesting that pronounced DEG patterns reflect meaningful functional responses. However, additional functional analyses are necessary to verify the biological relevance of the transcriptomic data.

Finally, it will be essential to explore the role of specific active components within the extracts. Which compounds identified in the UPLC–MS/MS analysis (Sayed et al. 2022) are responsible for the observed transcriptional and phenotypic effects, and do they act independently, additively, synergistically, or even antagonistically? To address these questions, single and combined components should be tested in both phenotypic and transcriptomic assays.

## Supplementary Information

Below is the link to the electronic supplementary material.

Supplementary file1 (PDF 436 KB)

Supplementary file2 (XLSX 3730 KB)

Supplementary file3 (XLSX 84 KB)

## Data Availability

All data generated or analyzed during this study are included in this published article, its supplementary data files or are accessible through GEO Series accession number GSE274478 (https://www.ncbi.nlm.nih.gov/geo/query/acc.cgi?acc=GSE274478).

## Declarations

### Clinical trial number

Clinical trial number: not applicable.
