# Supplementary material for: Transcriptomic insights into the healthspan-enhancing effects of C. chinensis seed and E. ulmoides bark extracts in Caenorhabditis elegans
Source: Biogerontology. 2025 Nov 13;26(6):203. doi: 10.1007/s10522-025-10349-1 (PMC12615526; doi:10.1007/s10522-025-10349-1)
Supplement: Supplementary file 1 — Supplementary file1 (PDF 436 KB) [file 10522_2025_10349_MOESM1_ESM.pdf]

Supplementary data S1-S9

Article:
Transcriptomic Insights into the Healthspan-Enhancing Effects of *C. chinensis* Seed and *E. ulmoides* Bark Extracts in *Caenorhabditis elegans*

Journal:
Biogerontology

Authors:
Shimaa M. A. Sayed, Anna Pitas, Christian Schmitz-Linneweber, Nadine Saul

Corresponding Author:
Nadine Saul
Affiliation: Molecular Genetics Group, Institute of Biology, Faculty of Life Sciences, Humboldt University of Berlin, Philippstr. 13, 10115 Berlin, Germany
e-mail: nadine.saul@hu-berlin.de

S1: List of used primers

Primer		5'→3' sequence	Ta	L	Efficiency (%)
act-1	Fw	TCCAAGAGAGGTATCCTTAC	56	169	98.3
	Rv	CGGTTAGCCTTTGGATTGAG			
cdc-42	Fw	ATTACGCCGTCACAGTAATG	56	248	93.7
	Rv	ATCCCTGAGATCGACTTGAG			
hsp-16.1	Fw	CTATTTCCGTCCAGCTCAAC	54.8	410	95.2
	Rv	TTTGTTCAACGGGCGCTTGC			
hsp-16.2	Fw	ACGCCAATTTGCTCCAGTCT	55	347	99.7
	Rv	TCTCTTCGACGATTGCCTGT			
hsp-70	Fw	AGCCGGTTGAAAAGGCACT	55	338	97.4
	Rv	TGTTTTGGAAGCTTTGGCAGG			
daf-21	Fw	ATTCGCTACCAGGCACTCAC	55.9	240	101.7
	Rv	GAATCCGACTCCGAACTGAC			
hsp-12-6	Fw	TGGAGTTGTCAATGTCCTCG	53.6	235	100.3
	Rv	TCCATGTGAATCCAAGTTGCTC			
lys-7	Fw	GGTTCCCCCGATTGTTGACT	56	206	101.9
	Rv	ATCCTTGTCCTGCTGGGTTG			
lys-1	Fw	TGGATTCAGGTTACCTCCCC	54.8	220	95
	Rv	TCAGTCCGTATTGCTTGGCT			
ins-23	Fw	AGCTTGGGAGAAAAGGCTGA	55	154	103
	Rv	TAAAGACCACGTCCTCCAGC			
far-3	Fw	AGCTTGGGAGAAAAGGCTGA	55	133	97.5
	Rv	TAAAGACCACGTCCTCCAGC			
far-3 RNAi	Fw	GCGGTTCTGCAGCTGCTTGGTTCTTCTCCGTT	55	485	102
	Rv	GCCAAACCGCGGCCTCCAAAAACTTGGCGACT			
M13-24F-BLUE (LGC)	Fw	GTAAAACGACGGCCAGTGAGCGCG	Used for sequencing company Biosearch Technologies, Berlin		
ASK-FN2 (LGC)	Rv	CGGCCTTTTTACGGTTCCTG	Used for sequencing company Biosearch Technologies, Berlin		

Ta: Annealing temperature [°C]; L: Product length [bp]

S2: qPCR protocol

Temperature	Minutes	Loops
95 °C	02:00	
95 °C	00:10	
Ta °C	00:20	35 cycles
72 °C	00:30	
95 °C	01:00	
Melting curve (Ta °C- 95 °C, steps 0.5 °C)	00:10	

Ta = Annealing temperature

S3: Construction of the *far-3* RNAi feeding strain

Primers were designed by using the National Center for Biotechnology Information (https://www.ncbi.nlm.nih.gov). In addition, the restriction sites Pst-I (forward) and Sac-II (reverse) and a few overhanging bases as "leader sequence" were added, to improve efficient cutting (www.addgene.org/protocols/pcr-cloning/). The primer pair (see primer pair "*far-3* RNAi" in Supplementary Table S1) was checked for the optimal annealing temperature (New England BioLabs TM Calculator) and the formation of dimers and hairpins. The estimated fragment size was 485 bp long. Primers were then ordered at Thermo Fisher Scientific and 100 µM stock solutions were stored at -80 °C.

To amplify part of the *far-3* sequence, the master mix shown in Table S3 A was used. 5 µl of water (as negative control) or 5 µl of gDNA was pipetted into PCR tubes and 45 µl of the master mix was added into each tube on ice. The PCR was run according to the program shown in Table S3 B. The PCR product was checked by gel electrophoresis to verify the correct size. The PCR product was then cleaned up using the Monarch® PCR & DNA Cleanup Kit (5 µg) according to the manufacturer's manual. The DNA concentration and purity were finally determined via NanoDrop measurement.

S3A: Reagents and volumes for the PCR master mix to amplify *far-3*

Reagent	volume [µl] for 1 sample
ddH_2O	36
Q5Buffer B (10x)	5
dNTP Mix (10 mM)	1
far-3 forward primer (10 µM)	1.25
far-3 reverse primer (10 µM)	1.25
Q5 polymerase	0.5
**Total volume**	45

S3B: PCR program to amplify *far-3*

Temperature	Minutes	Loops
95 °C	01:00	
95 °C	00:20	
53 °C	00:30	3 cycles
72 °C	01:00	
95 °C	00:20	
70 °C	00:30	35 cycles
72 °C	01:00	
72 °C	02:00	
15 °C	Hold	

S4: NCBI BLAST results of obtained sequences from the self-constructed *far-3* RNAi feeding strain

Gene	Primer	Total Score	Query Cover	E Value	Percent Identity
far-3	M13-24F-BLUE	896	42 %	0	100
	ASK-FN2	896	41 %	0	100

S5: Thermocycler protocol for the synthesis of cDNA

Temperature	Minutes
25 °C	10
50 °C	30
85 °C	5

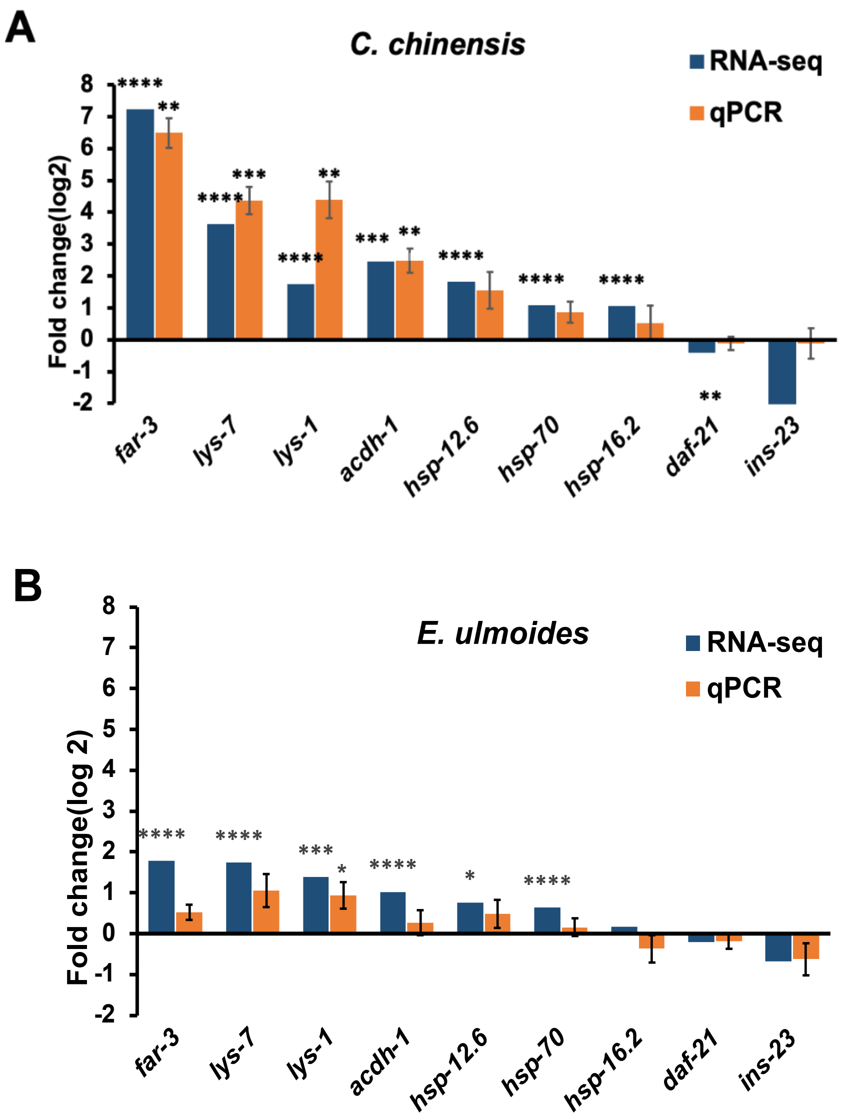

Figure S6: Validation of RNA-seq results using RT-qPCR. The expression profiles of eight genes in aged *C. elegans* after (A) *C. chinensis* or (B) *E. ulmoides* treatment were analyzed via RT-qPCR and the resulting expression values were calculated according to the Pfaffl method. The expression values of *act-1* and *cdc-42* were used to normalize the data. The bars represent the mean of three biological replicates. The error bars represent the standard error of the mean (SEM). The p-values are indicated by*(p < 0.05), ** (p < 0.01), *** (p < 0.001), or **** (p < 0.0001).

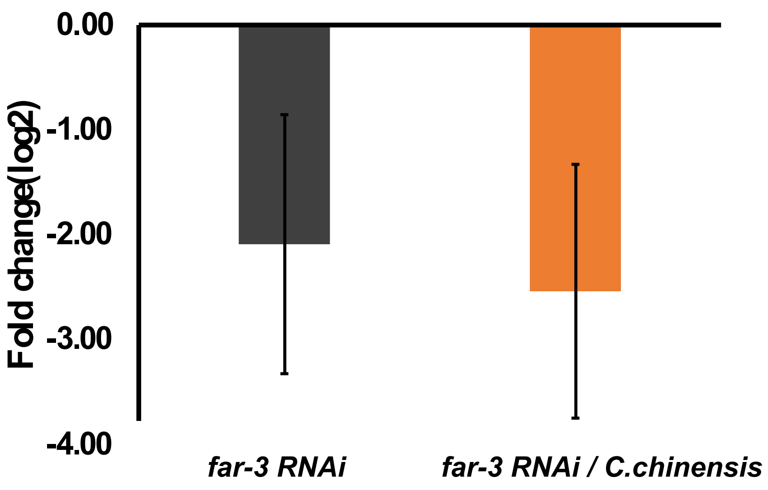

Figure S7: log2foldchange values for *far-3* after RNAi treatment. Worms were grown until the 3rd day of adulthood, and RNA interference was triggered from the L4 larvae stage via *E. coli* HT115 bacteria-feeding strains. Additionally, one group (orange bar) was also treated with *C. chinensis* extract in parallel. The expression values were calculated according to Pfaffl (2001). Gene expression values of the reference genes *act-1* and *cdc-42* were used to normalize the data. The graph of the RT-qPCR shows the mean of three biological replicates. The error bars show the standard error of the mean (SEM), and statistical significance was determined according to one-way ANOVA and post-hoc Bonferroni test with *($p < 0.05$).

S8: Lifespan analysis of *C. elegans* treated with either 30 µg/mL *C. chinensis/far-3* RNAi or only *far-3* RNAi, using EV RNAi for the control.

Treatment	Mean Lifespan		Min. (days)	Med. (days)	Max. (days)	n	p_{Bonf}
	Days ± SE	%					
Control	18.54 ± 0.34	100.0	15.47	17.88	25	219	
C. chinensis/far-3	19.06 ± 0.32	102.8	15.41	18.58	27	226	0.2966
far-3	17.92 ± 0.32	96.7	14.85	17.06	24	224	0.1371

SE = Standard error of the mean; Min/Med/Max lifespan = days till deaths in population reached 25%/50%/100%; The p-values (p_{Bonf}) represent significance determined by a log-rank test with *post-hoc* Bonferroni adjustment.

S9: Survival after heat stress of *C. elegans* on the 12th day of adulthood treated with either 30 µg/mL *C. chinensis/far-3* RNAi or only *far-3* RNAi, using EV RNAi for the control.

Treatment	Mean Survival		Min. (days)	Med. (days)	Max. (days)	n	p_{Bonf}
	Days ± SE	%					
Control	3.51 ± 0.1	100.0	2.11	2.96	8	219	
C. chinensis/far-3	3.26 ± 0.1	92.8	1.69	2.53	9	224	0.2569
far-3	3.39 ± 0.09	96.6	1.94	2.74	8	226	0.678

SE = Standard error of the mean; Min/Med/Max lifespan = days till deaths in population reached 25%/50%/100%; The p-values (p_{Bonf}) represent significance determined by a log-rank test with *post-hoc* Bonferroni adjustment.
